# Overcoming rheumatoid arthritis challenges: Ensuring timely referral to rheumatologists in resource-scarce countries

**DOI:** 10.2478/rir-2023-0033

**Published:** 2023-12-19

**Authors:** Anum Khan, Babur Salim, Shahida Perveen, Saba Samreen, Haris Gul, Amjad Nasim

**Affiliations:** Rheumatology Department, Fauji Foundation Hospital, Rawalpindi, Pakistan; Foundation University School of Health Sciences (FUSH), Rawalpindi, Pakistan

Dear Editor,

Rheumatoid arthritis (RA) is one of the most common autoimmune inflammatory diseases with an estimated prevalence of 0.5%–1%.^[1]^ Inflammation of the synovium is the hallmark of this disease, which can cause destruction and deformities of joints if left uncontrolled. Around 70% of the radiographic damage and deformities occur within the first two years of its onset, leading to permanent disability.^[2]^ Delaying treatment for more than a year has a significant association with radiographic damage.^[3]^

Early diagnosis and treatment are the essence of management of RA.^[4,5]^ Timely management increases the odds of a favorable outcome in RA patients by preserving functionality and reducing morbidity.

Local data of Pakistan depicts a mean delay in diagnosis of rheumatoid arthritis by 1 year, and therapeutic delay of 1.5 year.^[4]^ Rheumatologists believe that there is a “window of opportunity” for treating RA, that is early in the disease, which if availed results in a better prognosis and quality of life.^[6]^ Multiple factors are attributable to the diagnostic and therapeutic delay and losing the window of opportunity, such as lack of awareness and resources, diagnostic ambiguity in some cases of early RA and difficult access to a rheumatologist.

Considering the diagnostic and therapeutic difficulties, recent guidelines endorse that rheumatologists should treat patients with RA.^[5]^ This is hard to implement, as the prevalence of rheumatoid arthritis is 1% of the world’s population,^[7]^ and 0.55% of Pakistan’s population (1.15 million people in 2023)^[4]^ Against this burden of disease, the number of rheumatologists in Pakistan is limited, around 100 registered with Pakistan society of Rheumatology (PSR). These hurdles can be overcome by close collaboration of rheumatologists with other clinicians.

We are proposing a simple algorithm for general physicians to help them in diagnosis and management of early RA and when to refer a patient to a rheumatologist. The algorithm was created through consensus and voting of rheumatolo-gists in various cities of Pakistan. The selected items were finalized after a survey carried out among the general physicians during the project “Rheumatoid Arthritis Awareness Among Local General Physicians (GPs) of Pakistan: From Diagnosis to Referral” carried out by rheumatology department Fauji Foundation Hospital, funded by the International League of Associations for Rheumatology (ILAR) in 2023. This can refine the referral system and is easily applicable worldwide especially in countries where limited number of rheumatologists are available. The rationale of this referral is to diagnose and initiate early treatment. It should be noted that imaging has not been mentioned in this algorithm as it is a specialized tool used specifically by rheumatologists or radiologists (Figure 1). General physicians should know the following facts before referring an RA patient to a rheumatologist.

**Figure 1:**
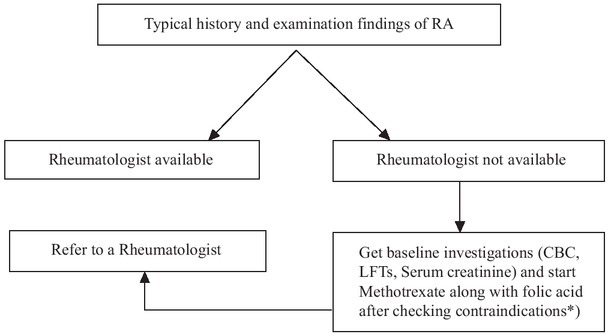
Rationale: Earlier initiation of optimal treatment. *Hypersensitivity, impaired liver function, pregnancy, breast-feeding, alcohol use, immunodeficiency syndromes, blood dyscrasias, severe renal impairment. CBC: complete blood count; LFTs: liver function tests.

## Picking arthritis/synovitis

**Figure 2:**
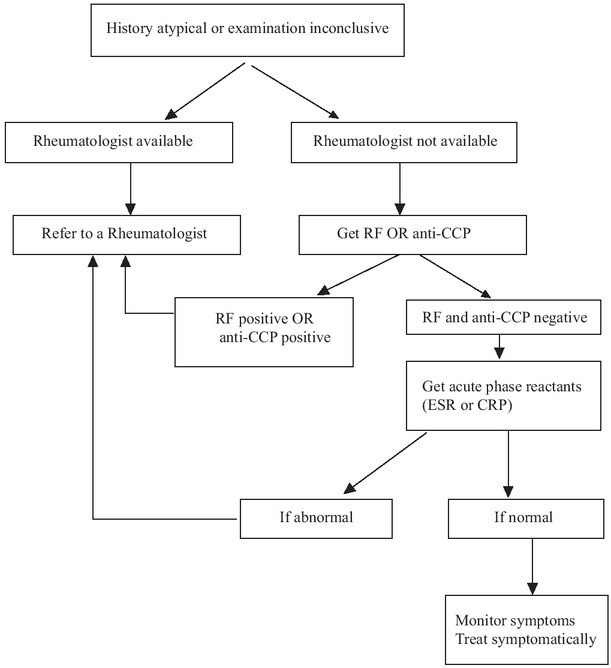
Approach for atypical cases. RF: rheumatoid factor; anti-CCP: anti-cyclic citrullinated peptide antibody; ESR: erythrocyte sedimentation rate; CRP: C-reactive protein.

Learn to pick arthritis/synovitis with history (joint swelling, pain, significant stiffness for at least an hour) and clinical examination (joint swelling, tenderness, limited range of motion). Rule out other causes of synovitis and polyarthritis on the basis of clinical history and examination, like viral (duration < 6 weeks), psoriasis (own or family history of skin or nail disease), systemic lupus erythematosus (presence of oral/nasal ulcers, alopecia, photosensitivity, rash, Raynaud’s phenomenon, muscle weakness/pain), crystal arthritis and nodal/erosive osteoarthritis *etc*. If there is suspicion of an alternate diagnosis, consider referral to a rheumatologist (Figure 2).

## Interpretation of laboratory tests

Laboratory tests like rheumatoid factor (RF), anti-cyclic citrul-linated peptide antibodies (anti-CCP), erythrocyte sedimentation rate (ESR) and C-reactive protein (CRP) can aid in diagnosing RA. Some laboratories in the peripheries don’t have the facility for testing anti-CCP and CRP, so either one of raised ESR or CRP, and RF or anti-CCP positivity can suffice. The diagnosis of rheumatoid arthritis is always clinical and never solely based on the positivity of tests. Around 70% of RA patients are RF and/or anti-CCP antibodies (seroposi-tive).^[8]^ In the presence of clinical features of RA and negative antibodies, it is called ‘seronegative RA’. Acute phase reactants (ESR and CRP) are non-specific and can be normal in up to 30% of patients with active rheumatoid arthritis.^[9]^ Therefore, interpret the tests judiciously.

## Base line tests prior to initiation of standard treatment

When the diagnosis of RA is established, it is essential to obtain complete blood count, liver function tests and serum creatinine before the initiation of disease modifying agent.

## Treatment

After confirming the diagnosis of RA, initiate treatment promptly with a conventional synthetic disease modifying anti-rheumatic drug (csDMARD). Methotrexate is the first line treatment drug unless there is any contraindication.^[2,5]^ Initiate at a dose of 7.5–15 mg/week and titrate dose up to 20–25 mg/week within 4–6 weeks after checking complete blood count (CBC), serum alanine transaminase (ALT) and creatinine. Folic acid must be co-prescribed with methotrexate to avoid its side effects (at least 5 mg/week). Consider oral short-term low-dose steroids (≤7.5 mg/day prednisone equivalent for about 3 months)^[5]^ as bridging therapy while the csDMARD takes effect.

## When to refer to a rheumatologist?

Ideally, rheumatologists are the primary physicians to treat RA patients, and the earlier the referral, the better the outcome. However, if the rheumatologist is not easily available or patient cannot reach the rheumatologist in time, start methotrex-ate (if there is no contraindication) considering early initiation of disease modifying drug can prevent irreversible damage. (1) Diagnosis is uncertain; (2) Severe or rapidly progressing disease; (3) Poor response to initial treatment within 3–4 months; (4) Life-threatening or organ-threatening extra-articular manifestations of RA (like vasculitis); (5) Pregnancy or fertility issues; (6) Need for biologic or targeted synthetic DMARD therapy.

## Follow up

We suggest to regularly monitor patient’s disease activity, response to treatment and any potential adverse effects, and collaborate with rheumatologist for long-term management plan.
